# Healthy Aging and Dementia: Two Roads Diverging in Midlife?

**DOI:** 10.3389/fnagi.2018.00275

**Published:** 2018-09-19

**Authors:** Katie Irwin, Claire Sexton, Tarun Daniel, Brian Lawlor, Lorina Naci

**Affiliations:** ^1^Department of Neuroscience, University of Georgia, Athens, GA, United States; ^2^Memory and Aging Center, Global Brain Health Institute, University of California, San Francisco, San Francisco, CA, United States; ^3^Oxford Centre for Human Brain Activity, Wellcome Centre for Integrative Neuroimaging, Department of Psychiatry, University of Oxford, Oxford, United Kingdom; ^4^Mercer’s Institute for Successful Ageing, St. James’s Hospital, Dublin, Ireland; ^5^The Global Brain Health Institute, Trinity College Dublin, Dublin, Ireland; ^6^School of Psychology, Trinity College Dublin, Dublin, Ireland

**Keywords:** aging, early markers, midlife, Alzheimer’s disease, dementia, cognition and brain structure

## Abstract

Dementia, particularly Alzheimer’s disease (AD), is a growing pandemic that presents profound challenges to healthcare systems, families, and societies throughout the world. By 2050, the number of people living with dementia worldwide could almost triple, from 47 to 132 million, with associated costs rising to $3 trillion. To reduce the future incidence of dementia, there is an immediate need for interventions that target the disease process from its earliest stages. Research programs are increasingly starting to focus on midlife as a critical period for the beginning of AD-related pathology, yet the indicators of the incipient disease process in asymptomatic individuals remain poorly understood. We address this important knowledge gap by examining evidence for cognitive and structural brain changes that may differentiate, from midlife, healthy aging and pathological AD-related processes. This review crystallizes emerging trends for divergence between the two and highlights current limitations and opportunities for future research in this area.

## Introduction

As more individuals are living longer than ever before, the older population is growing exponentially across the globe. In 2015, approximately 8.5% of the world’s population was over the age of 65, and by 2050, this number is expected to almost double to 16.7% (He et al., 2016). Understanding how to effectively promote healthy aging and prevent neurodegenerative diseases that present symptoms in older adults thus is becoming increasingly important.

One of the biggest challenges associated with the aging population is dementia. The World Alzheimer Report has suggested that the number of people living with dementia could triple to 132 million people worldwide by 2050 (Prince et al., 2015). Dementia not only dramatically alters the lives of those with the condition, but it also confers a severe burden on families, friends, caregivers, and the healthcare systems at large. The worldwide cost of dementia was estimated at $818 billion in 2016 and could increase to approximately $2 trillion by 2030 (Prince et al., 2015).

## What Is Dementia?

Although dementia predominantly affects older people, it is neither a normal part of aging nor an exaggeration of it. It is not a single disease, but instead a clinical syndrome formed by a range of recognizable symptoms. The Diagnostic and Statistical Manual of Mental Disorders (2013) Fifth Edition (DSM-5,), replaces the term “dementia” with “major neurocognitive disorder” and defines the condition as significant cognitive decline that, critical to the diagnosis, impairs independent living. Cognitive domains that can be impacted include learning and memory, executive function, language, complex attention, perceptual-motor function, and social cognition (Diagnostic and Statistical Manual of Mental Disorders, 2013). Dementia involves a myriad of adverse neurocognitive changes, and the precise pathological processes underlying these changes are not well understood (Sacuiu, 2016). This review focuses on AD, the most common type of dementia (Barker et al., 2002).

## Alzheimer’s Disease

Alzheimer’s disease accounts for around 60–80% of dementia cases, and its symptoms are projected to affect greater numbers of people every year (Alzheimer’s Association, 2017). Insidious and irreversible memory decline is the most recognized feature of AD, beginning with initial short-term memory deficits that make learning new information difficult, but other areas of cognition such as word-finding, visuoconstructional, and executive abilities can also decline (Sacuiu, 2016). As a patient progresses through mild, moderate, and severe stages of AD, greater memory deficits, increased confusion, and personality and behavioral changes, among other symptoms, are frequently observed and lead to round-the-clock assistance needs with everyday activities (Logiudice and Watson, 2014).

The precise brain mechanism affected by neural degeneration in the earliest stages of AD is still largely hypothesized (Sacuiu, 2016). Recent evidence suggests that various subcortical brain nuclei may show the first AD-related pathology (Stratmann et al., 2016). The transentorhinal region is thought to be the first affected site in the cerebral cortex, and in later stages of the disease, atrophy spreads throughout cerebral cortex association areas (Braak et al., 2006).

Alzheimer’s disease can be divided into two types: familial and sporadic, both of which are associated with similar symptoms. Less than 1% of cases are attributable to familial AD (Bateman et al., 2010), which is passed on from parents to children through mutations in specific genes (Bateman et al., 2010) and is associated with an earlier onset compared with sporadic AD (typically between ages 50 and 65, compared with after age 65 in sporadic AD). In contrast, sporadic AD does not have a specific genetic foundation (Piaceri et al., 2013). Instead, “susceptibility genes” that do not directly cause AD but increase one’s susceptibility of developing the disease are implicated. The most potent of the susceptibility genes is the APOE 𝜀4 allele (Corder et al., 1993). Carrying the 𝜀4 allele imposes a greater risk of developing AD compared to the 𝜀3 allele, increasing in a dose dependent manner from 20 to 90% in homozygous carriers (Corder et al., 1993), whereas the 𝜀2 allele has been suggested to provide a protective effect, lowering the risk of AD (Corder et al., 1994). For this reason, the APOE 𝜀4 allele is frequently used in studies as a proxy for the eventual diagnosis of AD, due to the difficulty of following up participants until the clinical diagnosis of AD is confirmed. However, because the APOE 𝜀4 allele is merely a risk factor, individuals that carry the allele may never develop AD, and, conversely, individuals without the allele can develop the disease (Michaelson, 2014). Paradoxically, the APOE 𝜀4 allele has been associated with better episodic memory in young adults, and the underlying reasons for this switch are poorly understood (Mondadori et al., 2006). In addition to susceptibility genes, an array of genetic, environmental, and lifestyle factors can contribute to the development of AD (Livingston et al., 2017), but the exact etiology of the disease remains unknown (Piaceri et al., 2013).

The question of when and in what ways healthy aging diverges from the incipient AD remains poorly understood and the focus of active research (Ritchie et al., 2015; Mortamais et al., 2017), with very recent research suggesting that this divergence may be observed as early as midlife (Ritchie et al., 2017). The identification of pathological aging in midlife could be transformational. The brain is thought to be modifiable in neural and cognitive ways (Lachman et al., 2015), so early detection and intervention could lead to improved treatment and, ultimately, prevention of Alzheimer’s dementia.

## Divergence in Midlife

Before dementia’s symptoms occur, an intermediate stage of MCI, formally termed minor neurocognitive disorder by DSM-5 (Diagnostic and Statistical Manual of Mental Disorders, 2013), may occur. MCI can be a transitional stage between normal aging and dementia, but not all people who experience it will develop dementia. MCI is characterized by observable cognitive deficits that resemble, but are less severe than, those typical of different dementias. In order to be labeled MCI, cognitive deficits must be greater than would be expected from the individual’s age and education level but not significantly impair daily functioning as in dementia (Albert et al., 2011; Diagnostic and Statistical Manual of Mental Disorders, 2013). However, particularly in AD, pathophysiological processes leading to the disorder may have already begun an irreversible trajectory of neurodegeneration by the stage of MCI, as several studies suggest that dementia’s pathology may be present years or even decades before its clinical diagnosis (Braak and Braak, 1997; Jack et al., 2009; Ritchie et al., 2015). Intervention prior to the development of MCI thus may be necessary to significantly reduce dementia incidence. Consequently, research efforts are increasingly focusing on preclinical AD – those at risk based on genetic and/or family history factors, but not yet showing symptoms – in order to detect early biomarkers, monitor progression, and employ disease-modifying agents as they become available (Dubois et al., 2016). However, the early divergence of healthy and pathological aging remains elusive. To address this critical knowledge gap, this review examines current evidence for significant cognitive and neural changes in midlife that may distinguish healthy and pathological brain aging indicative of AD (**Tables 1–3**).

**Table 1:** Studies of healthy versus pathological aging.

	MCI/Progression to MCI	AD/Progression to AD	APOE 𝜀4 carrier	Family history of AD	APOE 𝜀4 carrier and family history	VL/VL *TOMM-40* (w/ APOE 𝜀3)
Memory deficits		Kawas et al., 2003^†^; Grober et al., 2008^†^;			Debette et al., 2009^∗^	
Executive function deficits		Grober et al., 2008^†^; Mistridis et al., 2015^†^; Rajan et al., 2015^†^		Debette et al., 2009^∗^		
Visuospatial/ navigational deficits		Johnson et al., 2009^†^; Laukka et al., 2012^†^	Espeseth et al., 2006^∗^	Ritchie et al., 2017^∗^		
Gray matter atrophy			Alexander et al., 2012^∗^			Johnson et al., 2011^∗^
White matter damage		Head et al., 2004^‡^; Zhang et al., 2009^∗^	Persson et al., 2006^∗^; Westlye et al., 2012^∗^; Adluru et al., 2014^∗^	Bendlin et al., 2010^∗^	Adluru et al., 2014^∗^	
Network-related volume loss	Fjell et al., 2010^∗^					
Hippocampal volume loss		Thompson et al., 2004^‡^				

*Progression to MCI/AD, Individual was cognitively normal at beginning of study and went on to develop MCI/AD at some point during follow-up; APOE 𝜀4, Apolipoprotein E 𝜀4 allele; *TOMM-40*, Translocase of the outer mitochondrial membrane poly-T polymorphism; VL, Very long poly-T allele (associated with earlier onset of AD); APOE 𝜀3, Apolipoprotein E 𝜀3 allele; ^∗^reports on preclinical dementia in midlife; ^†^reports on preclinical dementia, not specific to midlife; ^‡^shows divergence of healthy and pathological aging, one area promising for midlife effects.*

**Table 2:** Divergence of healthy and pathological aging in cognitive studies.

Population Type	Study type	Sample Size/Age Range	Type of measurement	Effect	Evidence for divergence	Reference
Progression to AD	Longitudinal	*n* = 92; ages 60+ (mean = 79.8, *SD* = 6.9)	Episodic memory	Decreased	Acceleration of decline in FCSRT performance 7 years before diagnosis	Grober et al., 2008^†^
Progression to AD vs. healthy aging	Longitudinal	*n* = 1425 (144 progressed to AD); ages 60+ (mean = 78.1 at last follow-up)	Episodic visual memory	Decreased	More errors on BVRT 15 years before diagnosis	Kawas et al., 2003^†^
APOE 𝜀4 carriers – parental AD vs. no parental AD	Cross-sectional	*n* = 165; mean age = ∼59	Verbal and visuospatial memory	Decreased	Significantly lower LM-d and VR-d scores in carriers with parental AD	Debette et al., 2009^∗^
Progression to AD vs. healthy aging	Longitudinal	*n* = 2125; ages 65+	Executive function	Decreased	Strong associations with decline on Symbol Digits Modalities Test 13.0–17.9 years before diagnosis	Rajan et al., 2015^†^
Progression to MCI/later AD vs. healthy aging	Longitudinal	*n* = 87 (27 MCI/AD, 60 remained healthy); mean age = 65.78 at first visit	Executive function	Decreased	Divergence in TMT-B and intrusion errors 2 years before MCI, 4.5 years before AD	Mistridis et al., 2015^†^
Progression to AD	Longitudinal	*n* = 92; ages 60+ (mean = 79.8, *SD* = 6.9)	Executive function	Decreased	Acceleration of decline in Category Fluency, Letter Fluency, and TMT-B 2.5–3 years before diagnosis	Grober et al., 2008^†^
Parental AD vs. no parental AD	Longitudinal	*n* = 717; mean age = 59	Executive function	Decreased	Worsening scores on TrB-TrA in individuals with parental AD	Debette et al., 2009^∗^
Parental dementia vs. no parental dementia (AD = 56, mixed AD = 20, vascular = 16, unknown = 11)	Cross-sectional	*n* = 210; ages 40–59	Visuospatial/ navigational ability	Decreased	Higher Dementia Risk Scores significantly associated with poorer visual recognition; Family History subgroup closer to dementia onset had lower visual working memory scores	Ritchie et al., 2017^∗^
			Episodic memory	No effect	No significant association found at preclinical stage	
Progression to AD vs. healthy aging	Longitudinal	*n* = 851; mean age for progressed group = 82.5, for stable group = 81.7	Visuospatial ability	Decreased	Significant acceleration of decline in *Block Design* from WAIS-R performance compared to normal aging curve 9 years before diagnosis	Laukka et al., 2012^†^
Progression to AD vs. healthy aging	Longitudinal	*n* = 444; ages 60+ (mean age for progressed group = 80.4, for stable group = 74.4)	Visuospatial ability	Decreased	Inflection point on Block Design, Digit Symbol, TMT-A, and BVRT performance 3 years before diagnosis	Johnson et al., 2009^†^
APOE 𝜀4 carriers vs. non-carriers	Cross-sectional	*n* = 230 (ages 53–64, *n* = 110; ages 65–75, *n* = 120)	Visuospatial attention	Decreased	Increased reaction time costs of invalid cuing in carriers	Espeseth et al., 2006^∗^
Older adults w/ range of cognitive status	Cross-sectional	*n* = 35; mean age = 71.7	Visuospatial ability	Associated with alERC volume loss	alERC shown to display earliest AD-related atrophy (Miller et al., 2015)	Yeung et al., 2017^†^

*Progression to AD/MCI: Individual was cognitively normal at beginning of study and went on to develop AD/MCI at some point during follow-up; FCSRT, Free and Cued Selective Reminding Test; APOE 𝜀4, Apolipoprotein E 𝜀4 allele; BVRT, Brief Visuospatial Memory Test; Parental AD/dementia: Family history of AD/dementia, with one or both of the individual’s parents having a diagnosis of AD/dementia; LM-d, Logical Memory test; VR-d, Visual Reproductions test; TMT-B, Trail Making Test B; MCI, Mild Cognitive Impairment; TrB-TrA, Trail Making Test B minus A; WAIS-R, Wechsler Adult Intelligence Scale-Revised; TMT-A, Trail Making Test A; alERC, Anterolateral entorhinal cortex; ^∗^reports on preclinical dementia in midlife; ^†^reports on preclinical dementia, not specific to midlife.*

**Table 3:** Divergence of healthy and pathological aging in structural neuroimaging studies.

Population Type	Study Type	Sample Size/Age Range	Type of Measurement	Areas Affected	Evidence for Divergence	Reference
APOE 𝜀4 carriers vs. non-carriers (all w/1st- or 2nd-degree family history of dementia)	Cross-sectional	*n*= 24; ages 26–45	Gray matter atrophyGray matter increase	Bilateral dorsolateral and medial frontal, anterior cingulate, parietal, and lateral temporal corticesBilateral cerebellar, middle occipital, bilateral thalamic, bilateral fusiform, right lingual gyri, and some bilateral hippocampal regions	Patterns seen in carriers compared to non-carriers	Alexander et al., 2012^∗^
Healthy APOE 𝜀3 homozygous adults (VL/VL vs. S/S vs. S/VL *TOMM40* poly-T lengths)	Cross-sectional	*n* = 117 (VL/VL = 35, S/S = 38, S/VL = 44); mean age = 55	Gray matter atrophy	Medial ventral precuneus, ventral posterior cingulate	Dose-dependent effects (greater atrophy per VL allele)	Johnson et al., 2011^∗^
Late middle-aged adults assessed for family history of parental AD (FH) and APOE 𝜀4 genotype	Cross-sectional	*n* = 343; ages 47–76	White matter damage	Genu of corpus callosum, superior longitudinal fasciculusUncinate fasciculus	Higher FA in FH+Lower axial diffusivity in FH+, APOE 𝜀4-; higher axial diffusivity in FH+, APOE 𝜀4+	Adluru et al., 2014^∗^
APOE 𝜀4/𝜀4 vs. 𝜀3/𝜀4 vs. 𝜀3/𝜀3	Cross-sectional	*n*= 60; ages 49–79	White matter damage	Posterior corpus callosum, occipito-frontal fasciculus, left hippocampus	Reduced FA in carriers compared to non-carriers (ages 49–64)	Persson et al., 2006^∗^
APOE 𝜀4 carriers vs. non-carriers	Cross-sectional	*n* = 203; ages 21.1–69.9 (mean = 47.6)	White matter damage	Basal temporal lobe, internal capsule, anterior parts of corpus callosum, forceps minor, superior longitudinal fasciculus, occipital and corticospinal pathways	Increased RD and MD in carriers compared to non-carriers	Westlye et al., 2012^∗^
Young adult vs. non-demented older adult vs. AD older adult	Cross-sectional	*n* = 75 (25 young, 25 non-demented older, 25 AD older); mean age young = 22, older = 77, AD older = 77)	White matter damage	Corpus callosum, lobar regions	Age affects in anterior white matter; AD-specific effects in posterior white matter	Head et al., 2004^‡^
Middle-aged adults assessed for parental family history (FH) of AD and APOE 𝜀4 genotype	Cross-sectional	*n* = 136; mean age FH+/𝜀4+ = 57.5, FH+/𝜀4- = 57.4, FH-/𝜀4+ = 55.4, FH-/𝜀4- = 58.4	White matter damage	Bilateral anterior corona radiata, left uncinate fasciculus/inferior fronto-occipital fasciculus, left superior corona radiata, left superior longitudinal fasciculus, left tapetum, bilateral posterior corona radiata, parts of the corpus callosum, right posterior cingulum, bilateral hippocampus	Reduced FA in FH+	Bendlin et al., 2010^∗^
Non-demented vs. AD late middle-aged adults	Cross-sectional	*n* = 37; mean age cognitively normal = 61.5, AD = 62.8	White matter damage	Left anterior cingulum, left posterior cingulum, bilateral descending cingulum, left uncinate tracts	Reduced FA in AD compared to cognitively normal	Zhang et al., 2009^∗^
Young adult vs. MCI vs. non-demented older adult vs. mild AD	Cross-sectional	*n* = 372; mean age young = 22.2, MCI = 61.4, non-demented older = 76.7, mild AD = 76.6	Network-related volume loss	Superior frontal cortices, putamen, caudateHippocampus; entorhinal, retrosplenial, posterior cingulate, precuneus cortices	Age-related changesAD-related changes	Fjell et al., 2010^∗^
Non-demented vs. AD older adults	Longitudinal	*n* = 26; mean age non-demented = 71.4, AD = 68.4	Volume loss	Right hippocampus Left hippocampus	–8.2% ± 2.6% per year in AD; -0.2 ± 1.2% per year in controls	Thompson et al., 2004^‡^
				Left hippocampus	–4.9% ± 1.8% per year in AD; -3.8 ± 1.6% per year in controls	

*APOE 𝜀4, Apolipoprotein E 𝜀4 allele; *TOMM-40*, Translocase of the outer mitochondrial membrane poly-T polymorphism; VL, Very long poly-T allele (associated with earlier onset of AD); S, Short poly-T allele; FH, Family history of parental AD, meaning one or both of the individual’s parents has a diagnosis of AD/dementia; FA, Fractional anisotropy; MD, Mean diffusivity; aMCI, Amnestic mild cognitive impairment; RD, Radial diffusivity; ^∗^reports on preclinical dementia in midlife; ^‡^ shows divergence of healthy and pathological aging, one area promising for midlife effects.*

## Literature Search Criteria

Online research for studies evaluating potential midlife cognitive and neurobiological markers of AD was conducted in PubMed, and a report of the group’s age range was required. Midlife was considered 40–65 years, inclusive. Populations representing pathological aging were defined as those who developed AD in longitudinal studies that recruited healthy populations and tracked who developed dementia, as well as at-risk groups in cross-sectional studies that compared low- and high-risk individuals, based on genetic and/or family history factors. Behavioral studies of cognition and structural neuroimaging studies investigating the underlying brain mechanisms were included. Task-based functional neuroimaging studies were excluded due to wide variation between tasks across studies. Studies of uncommon groups, e.g., women with diabetes, were also excluded. **Table 1** shows common cognitive and neuroimaging-based deviations from age-matched healthy controls reported in midlife populations at risk for AD pathology.

## Cognition

Cognitive assessments that can detect subtle changes before noticeable clinical symptoms appear may provide robust markers of pathological aging. However, little consensus has been reached regarding the best methods for detecting cognitive symptoms of preclinical AD in individuals who do not meet the threshold for MCI diagnosis (Albert et al., 2011). This difficulty is not only attributable to the subtlety of these changes (Mortamais et al., 2017), but also to the tendency of cognitive assessments to implicate more than one aspect of cognition (Mortamais et al., 2017) and to discrepancies between cross-sectional and longitudinal study procedures (Salthouse, 2014). Despite these obstacles, early deficits in some aspects of cognition have been pinpointed by multiple studies as potential indicators of divergence from healthy aging. **Table 2** shows a summary of the evidence for cognitive divergence between healthy and pathological aging in midlife populations.

Normal aging involves many cognitive changes, including variations in memory, executive function, reasoning, spatial abilities, attention, and language abilities (Blazer et al., 2015). Most aspects of memory show age-related decline, with decrements of varying intensity (Healey and Kahana, 2016). Semantic memory has been shown to improve until age 60, while episodic memory abilities stay roughly the same until 60–65 years (Rönnlund et al., 2005; Nyberg et al., 2012). A longitudinal study of midlife women ages 42–52 at entry showed that they experienced a mean decline in verbal episodic memory of 0.02 per year, or 2% of the mean baseline score in 10 years (Karlamangla et al., 2017). After about 60 years, both semantic and episodic memory face a sharp decrease (Rönnlund et al., 2005).

As episodic memory is thought to remain relatively steady until 60 years (Rönnlund et al., 2005), significant decline before the age of 60 could be indicative of pathological aging. Studies have proposed that episodic memory is a strong predictor of dementia, with decline more sizeable than that associated with the normal age-related processes detectable 7–15 years before diagnosis of AD (Kawas et al., 2003; Grober et al., 2008; Mortamais et al., 2017). Multiple studies have suggested that episodic memory decline is a marker of preclinical AD, but few studies have focused on preclinical AD specifically in midlife. One study that assessed middle-aged offspring of individuals with sporadic AD (mean age, ∼59) showed that the interaction of parental AD (one or more parents diagnosed with AD), with APOE 𝜀4 status was significantly associated with declines in verbal and visuospatial memory (Debette et al., 2009). More studies assessing middle-aged participants are needed to support these associations.

Executive function – an umbrella term for many high-order cognitive processes including memory, inhibition, task switching, etc. (Turner and Spreng, 2012) and, broadly speaking, the ability to self-regulate behavior – also shows an age-related decline. Rönnlund et al. (2007) measured executive function performance with the Tower of Hanoi task and showed a decline after 65 years that prevailed after adjustments for education and test-retest effects. Reasoning, one facet of executive function and self-regulation of behavior and actions, similarly declines with age (Blazer et al., 2015). Cognitive decline in executive function has also been linked more broadly with cognitive decline in intelligence (Salthouse et al., 2003).

Studies have not reached a consensus on when executive function declines in dementia. Rajan et al. (2015) suggested that executive dysfunction precedes memory loss 13 or more years before AD diagnosis, based on effect on the Symbol Digits Modalities Test, while Mistridis et al. (2015) posited that executive function declines 4.5 years before diagnosis, according to Trail Making Test B and intrusion errors. The Baltimore Longitudinal Study of Aging agreed more with the latter figure, showing acceleration of decline in executive function, as tested by Category Fluency, Letter Fluency, and Trail Making Test B, 2.5–3 years before diagnosis (Grober et al., 2008). Despite these inconsistencies, evidence suggests that executive dysfunction is detectable in preclinical AD and that significant impairment in executive function before the age of 65 would indicate a divergence from healthy aging. Similarly to episodic memory decline, executive dysfunction’s relevance to preclinical AD specifically in midlife has been less well studied. Debette et al. (2009) showed that middle-aged participants with parental AD (mean age, 59) were more likely to experience executive function decline, but additional studies are needed to provide a strong evidence base for this association.

Spatial ability — the ability to comprehend spatial relations between objects or images —also declines with age. Studies that compare older to younger participants have historically found that older individuals take longer to perform tasks that require a good grasp of spatial information (Bruin et al., 2016). Although spatial abilities are known to decline with age (Bruin et al., 2016), this decline is exacerbated in pathological aging. Ritchie et al. (2017), used COGNITO, an extensive neuropsychological battery, to evaluate various aspects of cognition in 40–59 year-olds and found that individuals at higher risk for AD, based on family history, showed significantly larger deficits in visuospatial and navigational tasks, but not episodic memory as may be expected. A study by Laukka et al. (2012) similarly found visuospatial ability to show significant acceleration of decline before episodic or semantic memory, at 9 years prior to AD diagnosis. A longitudinal study by Johnson et al. (2009) that analyzed cognitive decline from the age of 60 showed that visuospatial performance exhibited a sharp inflection point 3 years before AD diagnosis and declined with increasing rates afterward, whereas individuals who did not develop AD remained relatively stable in this domain. These studies provide evidence that differences in visuospatial abilities detected by Ritchie et al. (2017) in middle-aged individuals at higher risk for dementia hold when participants are studied until clinical diagnosis.

Also using a risk-related proxy for progression to AD, Espeseth et al. (2006) found slower reaction times in an attention task following invalid location cues in healthy middle-aged APOE 𝜀4 carriers compared to non-carriers (𝜀3/𝜀3 genotype), further associating deficits in visuospatial processing with a higher risk for AD. More longitudinal studies that assess cognition in middle-aged individuals and follow participants until older age to confirm progression/no progression to AD are needed, so that midlife data is not restricted to high risk vs. low risk comparisons. A recent study by Yeung et al. (2017) used an eye-tracking approach to measure decline in the visuospatial ability of configural processing, or attending to the arrangement of an object’s features, which the group found was strongly associated with volume loss in the alERC, a region shown to display early AD-related atrophy (Miller et al., 2015). The eye-tracking cognitive task was proposed as a sensitive and practical way to detect changes in alERC volume (Yeung et al., 2017), which has been suggested as a valuable biomarker of preclinical dementia in midlife (see neuroimaging section), but the eye-tracking task has not yet been employed for a middle-aged study group. Overall, decline of visuospatial ability on both memory and attention task performance has shown promise as a midlife indicator of pathological aging.

Different facets of attention exhibit distinct age-related patterns of change. Selective attention abilities deteriorate with age (McDowd and Shaw, 2000), but recent research suggests that this reduction is due to increased distractibility in other senses rather than mere decline of attention abilities (Hugenschmidt et al., 2009). Divided attention shows age-related deficits after 60 years, as attention-switching tasks tend to take longer as people age (Tsang and Shaner, 1998; Glisky, 2007). Changes in sustained attention with age are still debated, however, as sustained attention has been shown to increase, decrease, and stay the same in different studies likely due to methodological disparities across studies (Staub et al., 2013). Variances from these patterns that are clearly indicative of pathological aging have not yet been identified, besides the interaction between APOE 𝜀4 and visuospatial attention shown by Espeseth et al. (2006).

Finally, the effects of healthy aging on language abilities vary between components of language processing. When presented with word-finding tasks, older participants report more cases of the TOT effect, and the proportion of TOTs is reported to linearly increase with age (Shafto and Tyler, 2014). In addition to TOT states, picture-naming tasks have provided evidence for decline in language production abilities with increasing age (Nicholas et al., 1985; Ramsay et al., 1999; Goral, 2004). Older participants also, on average, produce simpler sentences and speak at a slower rate than younger participants (Shafto and Tyler, 2014). In contrast to these age-related declines in speech production, speech comprehension, including syntax and semantics, appears more robust due to distinct patterns of gray matter preservation/atrophy relative to those involved in speech production (Shafto and Tyler, 2014). Further research is needed to determine whether deviations from these established patterns can serve as early indicators of AD.

## Structural Neuroimaging

The structure of the brain undergoes significant changes with age. Postmortem studies have documented an array of age-related changes in brain morphology, including reduced brain weight and volume, ventricular and sulcal expansion, decreased synaptic density, loss of neuronal bodies and dendritic spines, loss of myelination, accumulation of lipofuscin, and thinning of cerebral vasculature (Raz and Rodrigue, 2006; Lindenberger, 2014).

*In vivo* structural neuroimaging plays an important role in identifying typical, age-related changes to brain structure and significant differences stemming from pathological processes. Evidence for differences in brain structure between healthy and pathological aging in midlife populations is summarized in **Table 3**. Below, we discuss current evidence for significant differences with respect to gray matter atrophy, white matter damage, network-related volume loss, and hippocampal volume loss.

Cortical gray matter density reductions over dorsal frontal and parietal association cortices have been shown to occur at a steady pace between the ages of 7 and 60 but slow down afterward (Sowell et al., 2003). Cortical gray matter volume in the posterior temporal region, however, appears to increase up to the age of 30 years and then rapidly decline (Sowell et al., 2003). Reduction of gray matter density relatively early in life may indirectly measure an increase in cerebral myelination, as brain weight is fairly stable until age 40 (Dekaban, 1978). Because gray matter density continues to decline into the 50s and white matter volume has peaked around the mid-40s (Bartzokis et al., 2001; Sowell et al., 2003), studies have suggested that the later loss may be due to neurodegeneration rather than an increase in myelination. A recent longitudinal study in healthy Chinese adults above the age of 55 found annual rates of gray matter decline (cm^3^/year) of -0.57/-0.29/-0.39/-0.16 in the frontal/parietal/temporal/occipital lobes, respectively (Leong et al., 2017). The frontal and parietal measurements generally agree with Western studies of adults aged 30–99 in one study, 59–85 in another, and 64–86 in a third, which have reported rates of -0.56 to -1.05/-0.21 to -0.90 in frontal/parietal lobes, but the temporal/occipital measurements were lower than the Western estimates of -0.43 to -0.55/-0.33 to -0.36 for these regions (Jernigan et al., 2001; Resnick et al., 2003; Driscoll et al., 2009). Together, these studies have suggested that annual decline is fastest in frontal lobes, with rates suggested to decelerate with increasing age, initially slowing around the age of 40 (Storsve et al., 2014).

Different patterns of gray matter atrophy are found in AD. In general, as aforementioned, healthy age-related processes in midlife manifest rapid gray matter reductions in frontal regions (Terribilli et al., 2011), but young adult to early middle-aged APOE 𝜀4 carriers (26–45 years old) have shown greater gray matter reductions in dorsolateral and medial prefrontal, parietal, and lateral temporal regions, concomitant with increases in other brain regions compared to non-carriers (**Table 3**) (Alexander et al., 2012). An interpretation of these relative increases is that they may serve to sustain normal cognition despite the gray matter reductions of the prefrontal, parietal, and temporal regions in APOE 𝜀4 carriers (Alexander et al., 2012).

Another study used a *TOMM-40* (translocase of the outer mitochondrial membrane) poly-T polymorphism, shown to predict age of onset of AD, instead of APOE as a genetic indicator of increased AD risk. APOE 𝜀3/𝜀4 heterozygotes have shown an association between a short (S) poly-T allele and later onset of AD and between a very long (VL) poly-T allele and earlier onset of AD (Johnson et al., 2011). This study found that healthy middle-aged adults (mean age, 55) with APOE 𝜀3 homozygous, VL/VL *TOMM-40* genotypes showed decreasing gray matter volume in the medial ventral precuneus and ventral posterior cingulate, with dose-dependent effects compared to S/VL and S/S *TOMM-40* groups (Johnson et al., 2011). These studies suggest that, in relation to AD risk-related genotypes, structural effects on gray matter are already observable by middle age, decades before potential diagnosis of AD.

White matter has also been found to decrease during healthy aging and to show distinct patterns of change in pathological aging. DTI studies that have investigated white matter FA and mean diffusivity measures of structural brain connectivity have suggested that decline in white matter microstructure begins between 18 and 50 years (Westlye et al., 2010; Bartzokis et al., 2012; Kochunov et al., 2012; Lebel et al., 2012; Sexton et al., 2014). The recent study of healthy Chinese adults (Leong et al., 2017) found similar patterns for the rate of white matter decline as for gray matter, with atrophy greatest in the frontal regions, followed by temporal, parietal, and occipital regions. Several studies have reported similar findings of age-related white matter reduction being predominant in frontal regions (Gunning-Dixon et al., 2009; Barrick et al., 2010; Bennett et al., 2010; Burzynska et al., 2010; Sexton et al., 2014; Bender and Raz, 2015; Bender et al., 2016), and it is thought to accelerate beginning by about age 50 (Sexton et al., 2014).

However, in pathological compared to healthy aging in late middle-aged adults, loss of white matter integrity is not foremost in anterior regions, but instead appears most pronounced in regions including the cingulum, corpus callosum, and superior longitudinal fasciculus (Persson et al., 2006; Gunning-Dixon et al., 2009; Adluru et al., 2014). A DTI study found significant FA differences between APOE 𝜀4 carriers and non-carriers ages 49–64 in the occipito-frontal fasciculus and the posterior portion of the corpus callosum, suggesting white matter integrity is compromised in APOE 𝜀4 carriers before the onset of AD (Persson et al., 2006). Another DTI study comparing white matter in middle-aged APOE 𝜀4 carriers and non-carriers (mean age, 47.6) significantly associated risk for AD with increased radial and mean diffusivity likewise in the corpus callosum and occipital pathways, among other regions (see **Table 3** for full results) (Westlye et al., 2012). Another study found significantly lower FA levels associated with parental family history of AD in cognitively healthy, middle-aged adults (mean age, 56.99), rather than with only APOE status. Effects of family history were shown in regions known to be affected in AD, such as the medial temporal lobe, with larger effects found on the left side of the brain (Bendlin et al., 2010). Some studies have supported the value of these susceptibility proxies by yielding similar patterns. For example, Head et al. (2004) demonstrated a dissociation between normal and pathological aging in a DTI study of young adults, cognitively healthy older adults, and older adults with AD, where age-associated decline in white matter integrity was apparent in anterior brain regions, while AD-related effects were observed on posterior fiber tracts. One study that compared late middle-aged cognitively normal (mean age, 61.5) and AD (mean age, 62.8) individuals, instead of using susceptibility proxies, found similar results, with AD significantly associated with lower FA values in the cingulum and left uncinate tracts (Zhang et al., 2009). Taken together the above findings offer some evidence that topographical distributions of gray and white matter atrophy provide insights into deviations from healthy aging in midlife, yet more studies that follow participants to clinical diagnosis of MCI or AD are needed to validate these potential early neurobiological markers (Sexton et al., 2011).

In addition to regional loss of gray and white matter integrity, network-level patterns of brain volume loss have been shown to differentiate between healthy and pathological aging. The study of healthy Chinese adults (Leong et al., 2017) showed cerebral volume loss of 0.56%/year, similarly to Western longitudinal studies’ rates of -0.5% (Jack et al., 2005)/0.55% (Enzinger et al., 2005). Although cerebral shrinkage is normal in aging, a study by Fjell et al. (2010) concluded that while volumetric reductions in the fronto-striatal executive network are typical of healthy aging, greater changes in the medial temporo-parietal episodic memory network are representative of AD or progression to AD (see **Table 3** for full results). This study used CSF tau measures of, on average, late middle-aged MCI individuals (mean age, 61.4) to validate MRI patterns gathered from healthy young (mean age, 22.2), healthy elderly (mean age, 76.7), and mild AD (mean age, 76.6) participants; only volume loss in the medial temporo-parietal network was associated with pathological levels of tau in the MCI patients. These results provide evidence for a detectable divergence between patterns of morphometric change in healthy vs. pathological aging. Because the AD-associated network atrophy resembles the spread of pathological tau in AD, this atrophy could be related to disease progression mechanisms, further suggesting the potential for network-related atrophy as a sensitive marker of midlife progression toward AD (Fjell et al., 2010).

Within the medial temporal lobe, volume loss associated with AD has been shown to begin in the alERC and progress medially through the rest of the entorhinal cortex to the hippocampus (Miller et al., 2015). In these regions normal aging can involve atrophy of 0.2–3.8% per year, whereas individuals with AD can experience rates of 4.9–8.2% (Thompson et al., 2004). Some variability has been reported within this range (Fraser et al., 2015; Leong et al., 2017), yet the age-related hippocampal volume reductions shown in several longitudinal studies (Raz et al., 2005; Fraser et al., 2015; Leong et al., 2017) fail to reach the high rates reported in AD (Lindenberger, 2014). Studies extending the analysis of such diverging atrophy rates to midlife are still needed. Interestingly, studies have shown that reduced hippocampal volume, a hallmark of AD, is associated with decreased vestibular function (Brandt et al., 2005). Compared to age-matched controls, individuals with AD have been shown to display worse vestibular function, which is related to visuospatial abilities, a cognitive domain mentioned above to be impacted more strongly in pathological than healthy aging (Harun et al., 2016). The vestibular system could thus be investigated as a link between these two neurobiological and cognitive markers of AD.

## Conclusion

In summary, associations have been found between higher risk for AD and greater midlife decline in episodic memory and executive function (Debette et al., 2009). Other evidence may suggest, however, that trends in visuospatial ability deficits more strongly differentiate healthy vs. pathological aging in midlife (Ritchie et al., 2017). Other cognitive domains such as attention and language abilities have not yet displayed substantial differences in middle-aged individuals of varying dementia risk.

In addition to cognitive markers, structural neuroimaging has shown diverging trends in gray matter reduction and loss of white matter integrity in healthy vs. pathological aging. Healthy aging is more strongly associated with decline in frontal regions (Terribilli et al., 2011), while middle-aged individuals more likely to develop AD have shown greater gray matter reductions in dorsolateral and medial prefrontal, parietal, and lateral temporal regions (Alexander et al., 2012) and have shown loss of white matter integrity in regions including the cingulum, corpus callosum, superior longitudinal fasciculus, and left uncinate fasciculus (Persson et al., 2006; Gunning-Dixon et al., 2009; Adluru et al., 2014). Additionally, midlife volumetric reductions in the fronto-striatal executive network seem to be a normal part of aging, while reductions in the medial temporo-parietal episodic memory network seem to indicate pathological aging (Fjell et al., 2010). Finally, entorhinal cortex and hippocampal atrophy rates appear to diverge in healthy and pathological brain aging (Raz et al., 2005; Lindenberger, 2014; Fraser et al., 2015; Leong et al., 2017), but it is not yet known if this divergence is relevant to midlife.

## Limitations and Future Directions

Various limitations hinder the identification of midlife cognitive and neurobiological markers of preclinical AD. Selection bias can interfere with the accurate interpretation of findings, as individuals who are very healthy may decline to participate in aging studies because they are too busy and individuals who are too unhealthy may not be able to participate (Minder et al., 2002). Socially isolated people and people without financial means are also likely underrepresented in studies (Ford et al., 2008). Additionally, the methodology of aging studies is challenged by individuals who enter a study as a cognitively normal participant but develop (unnoticed) cognitive impairment associated with preclinical AD during the study (Lezak et al., 2012). Further, cross-sectional and longitudinal studies present unique difficulties that make comparison of findings across them problematic. Cross-sectional studies are prone to cohort effects, in which potentially vast differences between cohorts can confound findings assumed to be the result of aging (Hedden and Gabrieli, 2004; Salthouse, 2014), and longitudinal studies are prone to practice effects, in which cognitive decline can be hidden by individuals’ improvement on tests that they have completed several times (Salthouse, 2010; Abner et al., 2012). Attrition that results in the healthiest, wealthiest, or most motivated participants remaining in the study to its conclusion can also bias longitudinal studies (Van Beijsterveldt et al., 2002). Additionally, as aforementioned, many studies use the presence of the APOE 𝜀4 allele as a proxy for the future diagnosis of dementia due to the difficulty of following up study participants over many years, possibly decades, to clinical diagnosis. Differences between measures gathered from high- and low-risk participants, such as gray matter volume, for example, thus are not synonymous with differences between individuals who progress to AD diagnosis and those who do not.

The development of early cognitive and neurobiological markers for preclinical AD in midlife would be greatly facilitated by standardization of cognitive and neuroimaging measures used across research groups. Although some cognitive trends have been found, these often do not accurately capture the individual aging process, which can differ considerably across people and fluctuate within the individual (Lindenberger, 2014). The development of assessments that have predictive value at the individual level is crucially important for the identification of individuals who can benefit from early interventions and preventative measures. Until more sensitive and specific cognitive assessments are developed, neurobiological markers may provide more promise for predicting AD development at an individual level. Longitudinal studies that confirm AD diagnosis rather than relying on risk-related proxies are more challenging and costlier to conduct, but they may be necessary for more finely pinpointing markers differentiating healthy and pathological brain aging in midlife. Additionally, as AD-related pathology has been shown to appear in subcortical nuclei before the hippocampus as previously mentioned (Braak et al., 2006; Stratmann et al., 2016), future neuroimaging studies could focus on diverging rates of volumetric reduction in these regions instead of only in the entorhinal cortex and hippocampus.

Although many challenges surround the study of healthy aging and AD, significant progress has been made in understanding potential neurobiological and cognitive markers of preclinical AD. Further research is needed in regards to midlife, however. The significance of developing sensitive markers for detecting healthy vs. AD-related changes in midlife remains paramount, as this period constitutes a highly favorable therapeutic window for reducing risk factors and introducing disease-modifying agents once they are developed.

## Author Contributions

KI and TD wrote the initial manuscript. CS and BL provided critical feedback. LN provided feedback, edited the manuscript, and oversaw the project.

## Conflict of Interest Statement

The authors declare that the research was conducted in the absence of any commercial or financial relationships that could be construed as a potential conflict of interest. The reviewer MG-R and handling Editor declared their shared affiliation at the time of the review.

## Abbreviations

AD: Alzheimer’s disease
alERC: anterolateral entorhinal cortex
APOE: Apolipoprotein E
DTI: diffusion tensor imaging
FA: fractional anisotropy
MCI: mild cognitive impairment
S/VL: short/very long poly-T allele
TOMM: translocase of the outer mitochondrial membrane
TOT: tip-of-the-tongue
